# Complete mitochondrial genome of *Neoconidiobolus thromboides* (*Entomophthorales*: *Ancylistaceae*)

**DOI:** 10.1080/23802359.2021.1934167

**Published:** 2021-06-03

**Authors:** Yong Nie, Zi-Min Wang, Heng Zhao, Xiao-Yong Liu, Bo Huang

**Affiliations:** a School of Civil Engineering and Architecture, Anhui University of Technology, Ma'anshan, China; b Anhui Provincial Key Laboratory for Microbial Pest Control, Anhui Agricultural University, Hefei, China; c State Key Laboratory of Mycology, Institute of Microbiology, Chinese Academy of Sciences, Beijing, China

**Keywords:** *Neoconidiobolus*, Basal fungi, Mitochondrial genome, Phylogeny

## Abstract

*Neoconidiobolus thromboides* is a pandemic species in the genus *Neoconidiobolus*. In this article, we report the first complete sequence of mitochondrial genome from a common entomophthoroid fungus *Neoconidiobolus thromboides* under Illumina next-generation sequencing system. The total length of the mitogenome is 34,984 bp with a GC content of 26.99%. The gene annotation revealed 56 genes, including 30 protein-coding genes (PCGs), two ribosomal RNA genes (rDNAs), 24 transfer RNA (tRNA) genes. Phylogenetic analyses of 14 concatenated conserved PCGs indicated that *N. thromboides* was grouped with *Capillidium heterosporum* and *Conidiobolus* sp.

The genus *Neoconidiobolus* accommodating all members of the traditional genus *Conidiobolus* subgenus *Conidiobolus* which produces neither microconidia nor capilliconidia was established recently (Nie et al. 2020). The nuclear genome of *N. thromboides*, one of the most common species in this genus, has been deposited in JGI (https://genome.jgi.doe.gov), but no mitochondrial genome information has been reported until now.

The ex-type strain ATCC 12587 of *N. thromboides* was obtained from American Type Culture Collection (Manassas, VA). An inoculum was incubated on PDA for 7 day at 21 °C, and genomic DNA was extracted from the mycelia using the CTAB method (Watanabe et al. 2010). The specimen number ATCC 12587 and the genomic DNA were deposited in the Research Center for Entomogenous Fungi (RCEF), Anhui Agricultural University, Hefei, Anhui, China (bhuang@ahau.edu.cn). The sequencing library was prepared according to the manufacturer’s instruction using the Paired-End DNA Sequencing Kit (Biooscientific, AIR™). The whole genomic sequencing (WGS) was performed by the Illumina HiSeq X Ten Platform (Pacific Biosciences, Nextomics Biosciences, Co., Ltd., Wuhan, China). After a quality control, the clean data was used to assemble the mitogenome by the software Norgal 1.0 (Al-Nakeeb et al. 2017). This mitogenome was annotated using the MFannot tool (http://megasun.bch.umontreal.ca/cgi-bin/mfannot/mfannotInterface.pl), and then manually corrected. The tRNA genes were predicted by tRNAscan-SE 1.3.1 (Lowe and Eddy 1997) based on the mitochondrial genetic code (genetic code 4) (Zhang et al. 2017; Nie et al. 2019).

The mitogenome of *Neoconidiobolus thromboides* (GenBank Accession no. MW795364) is 34,987 bp long with a GC content of 26.99%. It contains two ribosomal RNA genes (rnl and rns), 24 tRNA genes, and 30 protein-coding genes (PCGs). The PCGs include 14 standard ones of the electron transport and oxidative phosphorylation system, 15 free-standing ORFs, and 1 ribosomal protein S3 gene. Seven introns were found in PCGs, one each in *cox2*, *nad1* and *nad4*, and two each in *cob* and *cox1*.

The concatenated amino acid sequences of 14 PCGs were used for phylogenetic analyses. The sequences of 14 proteins were locally aligned with BioEdit (Hall 1999) and concatenated with SequenceMatrix (Vaidya et al. 2011). The maximum-likelihood (ML) tree was constructed using raxmlGUI 1.5b1 with GTRGAMMA substitution model (Silvestro and Michalak 2012). *Drosophila melanogaster* (NC_024511) and *Monosiga brevicollis* (NC_004309) were chosen as outgroups. In the clade of *Entomophthoromycotina* (Figure 1), *N. thromboides* was grouped with *Capillidium heterosporum* and *Conidiobolus* sp., which was congruent with previous studies (Nie et al. 2019; Sun et al. 2019). This result will provide an insight of phylogeny of *Neoconidiobolus* in the basal fungi.

**Figure 1.**
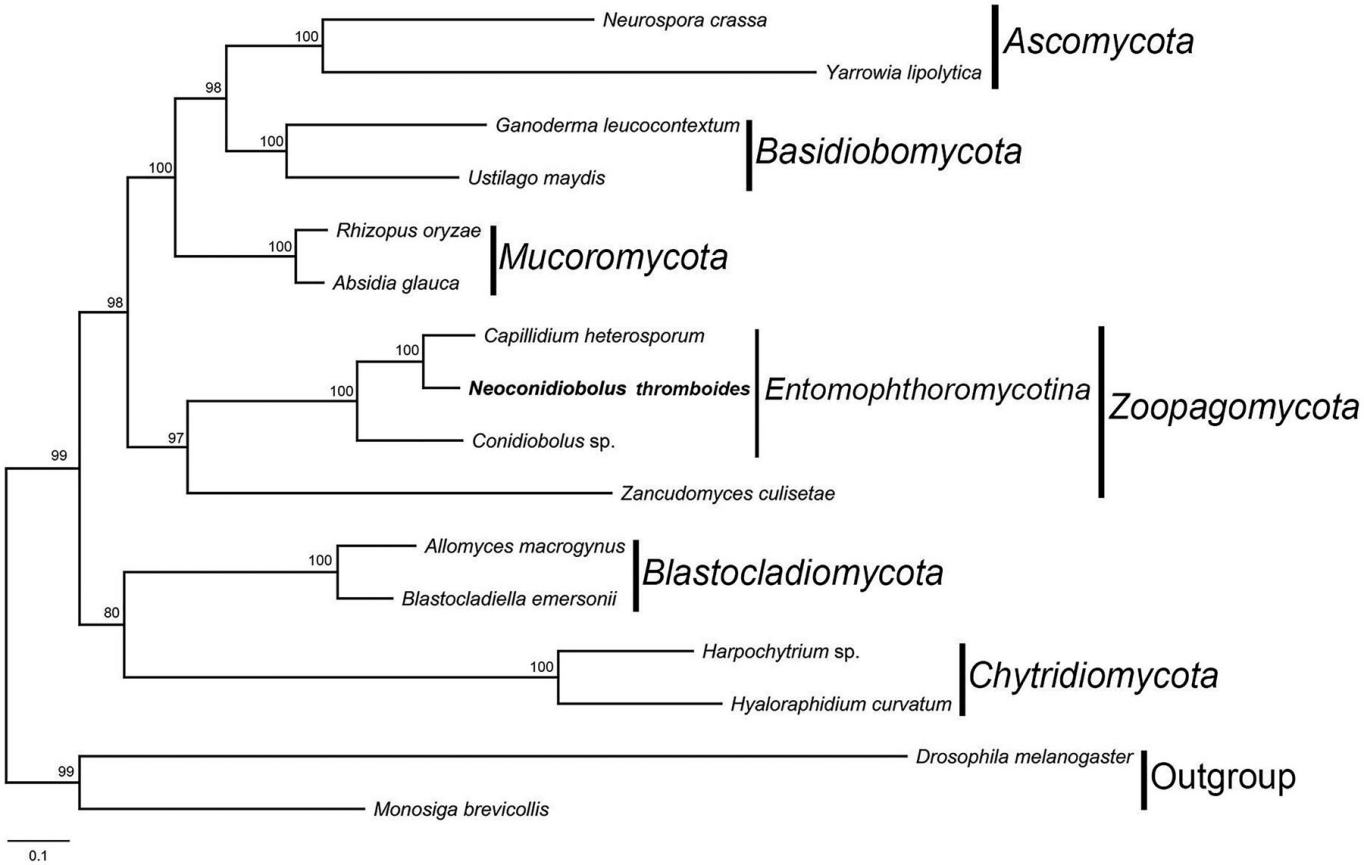
The phylogenetic tree of 14 fungal taxa constructed by maximum likelihood algorithm based on 14 translated mitochondrial proteins. These proteins include oxidase subunits (Cox1, 2, and 3), the apocytochrome b (Cob), ATP synthase subunits (Atp6, Atp8, and Atp9), NADH dehydrogenase subunits (Nad1, 2, 3, 4, 5, 6, and Nad4L). Along with the mitogenome of Neoconidiobolus thromboides, other 13 fungal mitogenomes were used in this phylogenetic analysis: *Absidia glauca* (NC_036158), *Allomyces macrogynus* (NC_001715), *Blastocladiella emersonii* (NC_011360), *Capillidium heterosporum* (NC_040967), *Conidiobolus* sp. (MN_640580), *Ganoderma leucocontextum* (NC_037937), *Harpochytrium* sp. (NC_004623), *Hyaloraphidium curvatum* (NC_003048), *Neurospora crassa* (NC_026614), *Rhizopus oryzae* (NC_006836), *Ustilago maydis* (NC_008368), *Yarrowia lipolytica* (NC_002659), and *Zancudomyces culisetae* (NC_006837). Besides, *Drosophila melanogaster* (NC_024511) and *Monosiga brevicollis* (NC_004309) were served as outgroups. Maximum-likelihood bootstrap values (500 replicates) of each clade are indicated along branches. Scale bar indicates substitutions per site.

## Data Availability

The data that support the findings of this study are openly available in GenBank of NCBI at https://www.ncbi.nlm.nih.gov, Reference no. MW795364.

## References

[CIT0001] Al-Nakeeb K, Petersen TN, Sicheritz-Pontén T. 2017. Norgal: extraction and de novo assembly of mitochondrial DNA from whole-genome sequencing data. BMC Bioinformatics. 18(1):510–517. 29162031 10.1186/s12859-017-1927-y PMC5699183

[CIT0002] Hall TA. 1999. BioEdit: a user-friendly biological sequence alignment editor and analysis program for Windows 95/98/NT. Nucleic Acids Symp Ser. 41:95–98.

[CIT0003] Lowe TM, Eddy SR. 1997. tRNAscan-SE: a program for improved detection of transfer RNA genes in genomic sequence. Nucleic Acids Res. 25(5):955–964. 9023104 10.1093/nar/25.5.955 PMC146525

[CIT0004] Nie Y, Wang L, Cai Y, Tao W, Zhang YJ, Huang B. 2019. Mitochondrial genome of the entomophthoroid fungus *Conidiobolus heterosporus* provides insights into evolution of basal fungi. Appl Microbiol Biotechnol. 103(3):1379–1391. 30569217 10.1007/s00253-018-9549-5

[CIT0005] Nie Y, Yu DS, Wang CF, Liu XY, Huang B. 2020. A taxonomic revision of the genus *Conidiobolus* (*Ancylistaceae*, *Entomophthorales*): four clades including three new genera. Mycokeys. 66:55–81. 32273794 10.3897/mycokeys.66.46575 PMC7136305

[CIT0006] Silvestro D, Michalak I. 2012. RaxmlGUI: a graphical front-end for RAxML. Org Divers Evol. 12(4):335–337.

[CIT0007] Sun XR, Su D, Gui WJ, Luo F, Chen Y. 2019. Characterization and phylogenetic analysis of the complete mitochondrial genome of *Conidiobolus* sp. (*Entomophthorales*: *Ancylistaceae*). Mitochondrial DNA Part B. 5(1):121–122. 33366449 10.1080/23802359.2019.1698340 PMC7721048

[CIT0008] Vaidya G, Lohman DJ, Meier R. 2011. SequenceMatrix: concatenation software for the fast assembly of multi-gene datasets with character set and codon information. Cladistics. 27(2):171–180. 10.1111/j.1096-0031.2010.00329.x 34875773

[CIT0009] Watanabe M, Lee K, Goto K, Kumagai S, Sugita-Konishi Y, Hara-Kudo Y. 2010. Rapid and effective DNA extraction method with bead grinding for a large amount of fungal DNA. J Food Prot. 73(6):1077–1084. 20537263 10.4315/0362-028x-73.6.1077

[CIT0010] Zhang YJ, Zhang HY, Liu XZ, Zhang S. 2017. Mitochondrial genome of the nematode endoparasitic fungus *Hirsutella vermicola* reveals a high level of synteny in the family *Ophiocordycipitaceae*. Appl Microbiol Biotechnol. 101(8):3295–3244. 28341884 10.1007/s00253-017-8257-x

